# Black Queen Evolution and Trophic Interactions Determine Plasmid Survival after the Disruption of the Conjugation Network

**DOI:** 10.1128/mSystems.00104-18

**Published:** 2018-10-02

**Authors:** Johannes Cairns, Katariina Koskinen, Reetta Penttinen, Tommi Patinen, Anna Hartikainen, Roosa Jokela, Liisa Ruusulehto, Sirja Viitamäki, Sari Mattila, Teppo Hiltunen, Matti Jalasvuori

**Affiliations:** a Department of Microbiology, University of Helsinki, Helsinki, Finland; b Department of Biological and Environmental Science, Centre of Excellence in Biological Interactions, Nanoscience Center, University of Jyväskylä, Jyväskylä, Finland; c Department of Biology, University of Turku, Turku, Finland; d Department of Genetics, University of Cambridge, Cambridge, United Kingdom; Dartmouth College

**Keywords:** antibiotic resistance, Black Queen evolution, conjugation, predation, trophic levels

## Abstract

Mobile genetic elements such as conjugative plasmids are responsible for antibiotic resistance phenotypes in many bacterial pathogens. The ability to conjugate, the presence of antibiotics, and ecological interactions all have a notable role in the persistence of plasmids in bacterial populations. Here, we set out to investigate the contribution of these factors when the conjugation network was disturbed by a plasmid-dependent bacteriophage. Phage alone effectively caused the population to lose plasmids, thus rendering them susceptible to antibiotics. Leakiness of the antibiotic resistance mechanism allowing Black Queen evolution (i.e. a “race to the bottom”) was a more significant factor than the antibiotic concentration (lethal vs sublethal) in determining plasmid prevalence. Interestingly, plasmid loss was also prevented by protozoan predation. These results show that outcomes of attempts to resensitize bacterial communities by disrupting the conjugation network are highly dependent on ecological factors and resistance mechanisms.

**IMPORTANCE** Bacterial antibiotic resistance is often a part of mobile genetic elements that move from one bacterium to another. By interfering with the horizontal movement and the maintenance of these elements, it is possible to remove the resistance from the population. Here, we show that a so-called plasmid-dependent bacteriophage causes the initially resistant bacterial population to become susceptible to antibiotics. However, this effect is efficiently countered when the system also contains a predator that feeds on bacteria. Moreover, when the environment contains antibiotics, the survival of resistance is dependent on the resistance mechanism. When bacteria can help their contemporaries to degrade antibiotics, resistance is maintained by only a fraction of the community. On the other hand, when bacteria cannot help others, then all bacteria remain resistant. The concentration of the antibiotic played a less notable role than the antibiotic used. This report shows that the survival of antibiotic resistance in bacterial communities represents a complex process where many factors present in real-life systems define whether or not resistance is actually lost.

## Abstract (precis)

Bacterial antibiotic resistance is often a part of mobile genetic elements that move from one bacterium to another. By interfering with the horizontal movement and the maintenance of these elements, it is possible to remove the resistance from the population. Here, we show that a so-called plasmid-dependent bacteriophage causes the initially resistant bacterial population to become susceptible to antibiotics. However, this effect is efficiently countered when the system also contains a predator that feeds on bacteria. Moreover, when the environment contains antibiotics, the survival of resistance is dependent on the resistance mechanism. When bacteria can help their contemporaries to degrade antibiotics, resistance is maintained by only a fraction of the community. On the other hand, when bacteria cannot help others, then all bacteria remain resistant. The concentration of the antibiotic played a less notable role than the antibiotic used. This report shows that the survival of antibiotic resistance in bacterial communities represents a complex process where many factors present in real-life systems define whether or not resistance is actually lost.

## INTRODUCTION

Bacterial resistance to antibiotics has emerged as a serious concern for modern health care. The majority of resistant bacteria in hospitals harbor mobile genetic elements that provide the bacteria with their efficient resistance phenotype. Therefore, the maintenance of resistance in a bacterial community is in many cases tightly linked to the survival of the mobile elements themselves (1). Horizontal gene transfer is a dominant feature among bacteria as environmental selection can favor individual organisms in a population that have either acquired or lost a particular gene. Studies have shown that genes are exchanged readily even between taxa also in cases where anthropogenic selection has induced a notable fitness benefit with respect to a particular trait (e.g., cephalosporin resistance) only relatively recently (2). As such, the microbiome appears to conform to a great extent to the Baas-Becking hypothesis that “everything is everywhere, but the environment selects.” In the case of resistance, the selection itself might appear to represent a relatively simple issue given that resistance should be beneficial only in the presence of antibiotics and costly to the bacterium in their absence. Based on this argument alone, the best solution to the resistance problem would be the careful stewardship of antibiotic use. However, in reality, the survival of resistance-conferring elements, such as conjugative plasmids, relies on various factors.

Bacterial hosts and their plasmids can coadapt, thus ameliorating plasmid-associated fitness costs (3–6). In Pseudomonas fluorescens, the underlying adaptive mutations in the host were shown to occur in the region encoding the bacterial *gacA*/*gacS* two-component global regulatory system (7). Mutations in the N terminus of the plasmid-encoded replication protein TrfA1 compensated for the cost of carrying a broad-host-range plasmid in Shewanella oneidensis (8). Loftie-Eaton and colleagues demonstrated that once a plasmid had acquired a transposon carrying a putative toxin-antitoxin system along with a resolvase gene, its persistence increased significantly in various hosts (5). The results of those recent studies imply that various adaptive changes in both the plasmid and the host can influence plasmid survival. However, pairwise adaptation itself is not likely to completely compensate for the costs, at least not in communities where the plasmid is continuously transferred into naive hosts. Therefore, interhost mobility itself is likely to play a role in plasmid maintenance (9), but the extent to which this is relevant has been debated (10–12). In a recent study, Lopatkin and colleagues conducted a meticulous analysis of the role of bacterial conjugation in maintaining resistance plasmids, showing that if the rate of plasmid loss and the costs of plasmid carriage are low enough compared to the frequency of horizontal transfer, then no selection (such as that associated with antibiotics) is required for plasmid survival (1). This was true also for communities consisting of several plasmids and bacterial strains, and it was therefore argued that disrupting conjugation and plasmid segregation can provide an avenue to limit the maintenance of resistance. Indeed, the presence of linoleic acid (inhibiting conjugation) and phenothiazine (promoting segregation loss) significantly reduced plasmid persistence in the community (1).

Naturally, plasmid survival in various environments (such as the human gut) is also heavily influenced by direct selection via antibiotics, since it is often impossible to completely avoid their administration. Antibiotic use results in environmental concentration gradients where they exert either inhibitory or subinhibitory effects on bacterial growth. In certain cases, plasmids may be transferred to susceptible but perhaps otherwise competitively superior hosts even after the initial exposure to a lethal antimicrobial component, thus restoring their positive growth (13). Further, subinhibitory concentrations can promote the maintenance of resistance plasmids by alleviating associated costs (14).

The “altruism” of the resistance mechanism also influences the selective landscape in the presence of antibiotics. Yurtsev and colleagues have shown that bacterial “cheaters” can stably persist in the presence of high concentrations of beta-lactam antibiotics provided that a fraction (depending on the initial concentration of antibiotic) still retains resistance (15). Similarly, altruism, or, more accurately, “leakiness,” of genetically encoded functions plays an important part in biology as various processes generate products that can be “public” (i.e. accessible by other organisms) (16). As the production of public goods is often costly to the producer, competition favors those that can rely on others to provide the necessary functions. Such examples of “Black Queen evolution” lead to the “race to the bottom,” that is, the loss of genes which are not needed (17, 18). The existence of gene-depleted “beneficiaries,” however, requires a fraction of the community to remain as “helpers.” Here, beta-lactam resistance in a mixed population is a prime example (15, 13). However, other resistance mechanisms, such as enzymatic resistance to aminoglycosides, cannot be readily exploited by those not producing the enzyme themselves. Therefore, in the presence of antibiotics, the possibility to race to the bottom is yet another facet that determines the extent to which resistance plasmids are maintained. It is also notable that in natural environments, bacteria do not exist only in communities with other bacteria, plasmids, and parasites such as bacteriophages but also with organisms that feed on them. Trophic interactions between bacteria and their consumers such as protozoa have received little attention in the research concerning resistance plasmid maintenance, which may partly result from their seemingly irrelevant effect on plasmids that reside within bacteria. Yet in natural microbial communities such as seas and freshwater systems, protozoa are considered to be major consumers of bacteria (19–21). They are present in wastewater treatment plants (22) and sometimes also exist in the human gut (23). Therefore, they can modulate the ecological landscape of (potential) plasmid hosts in many different types of environments. Recently, it was shown that, in contrast to a wild-type conjugative plasmid, a conjugation-deficient mutant was unable to persist in a bacterial population under conditions of predation (24). In contrast, in the absence of predation, the conjugation-defective mutant had higher persistence than the conjugative plasmid, suggesting that the relevance of conjugation to plasmid persistence may become evident only in a more realistic trophic setup.

**FIG 1:**
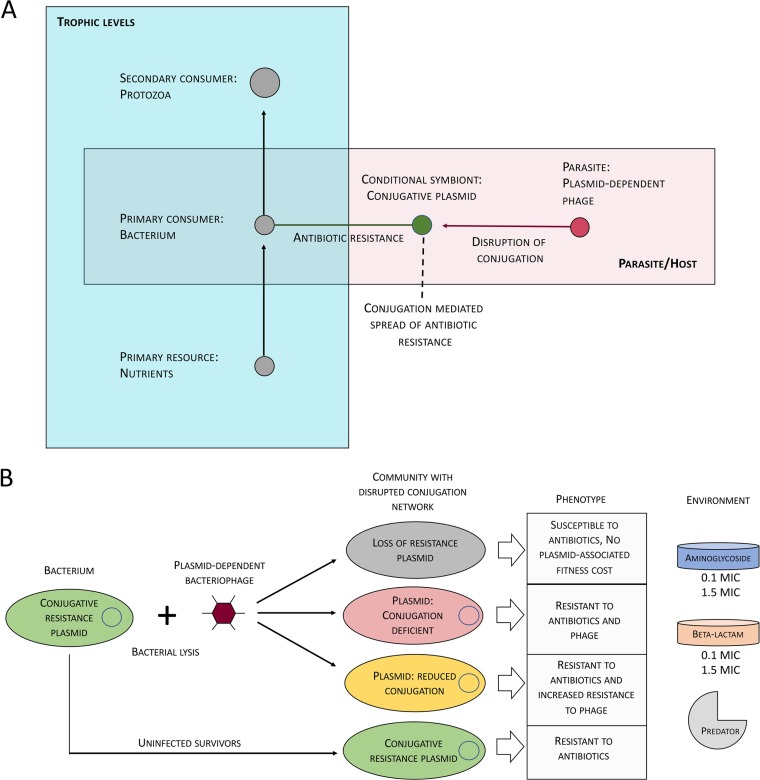
Schematic presentation of the ecological and evolutionary interactions investigated in the factorial experimental setup. (A) The trophic contacts in the community experiment. (B) Disruption of the conjugation network induced by the presence of plasmid-dependent bacteriophage and the factors in the experiments (protozoa, phage, two antibiotics with different resistance mechanisms).

Theoretical and experimental advances have been made to understand the dissection of populations into beneficiaries and helpers in the presence of different antibiotics and, together with the studies on the role of conjugative transfer in the maintenance of resistance plasmids, are starting to clarify the complexity behind the general antibiotic resistance problem. However, the interventions in the network that would resensitize communities to antibiotics are less extensively studied. Given that the effectiveness of the disrupted network is likely to depend on all the various factors described above, we set out here to use a factorial experimental setup to investigate the maintenance of resistance plasmids and conjugative phenotypes in a multitrophic community supplemented with antibiotics against which the plasmid encodes either “leaky” or “nonleaky” resistance (Fig. 1). For the initial disruption of plasmid conjugation, we utilized a natural “antiplasmid agent,” the bacteriophage PRD1, which specifically recognizes plasmid-encoded receptors on a bacterial cell. Exposing populations to PRD1 is known to select for cells that have lost either their plasmids (become resensitized) or, in part or completely, their conjugative ability (providing increased or full resistance to phage, respectively) (25, 26). In this study, we showed that two main factors have a major effect on plasmid prevalence: leakiness of antibiotic resistance and the modulation of the community by predation. These results suggest that even brief periods of exposure to low levels of antibiotics against which resistance is nonleaky can considerably increase the fraction of the population harboring a resistance plasmid. In contrast, with antibiotics against which resistance is leaky, such as beta-lactams, the effect is less notable even at inhibitory concentrations. Furthermore, the addition of a next level to the trophic network efficiently promoted plasmid persistence under all conditions. This was likely to result from lowered cell density, which in turn enhanced the per-cell metabolic activity and probably contributed to increased bacterial conjugation rates. Hence, these results give rise to the interesting possibility that protozoan predators may be playing a previously unrecognized role in promoting the prevalence of antibiotic resistance in various environmental reservoirs.

## RESULTS

### Community dynamics.

To understand the relative contributions of antibiotic concentration, altruism of resistance, and ecological interactions on plasmid persistence, we performed a 50-day microcosm experiment. We used a fully factorial experimental design with two treatments: (i) a community composition treatment, consisting of the bacterium Escherichia coli K-12 HMS174(RP4) harboring the multidrug (ampicillin, kanamycin, tetracycline) resistance plasmid RP4 with or without the plasmid-dependent bacteriophage PRD1 (hereafter, phage) and/or the ciliated protozoan Tetrahymena thermophila CCAP 1630/1U, and (ii) an antibiotic treatment consisting of no antibiotic or 0.1× MIC of ampicillin or kanamycin and 1.5× MIC of ampicillin or kanamycin against which RP4 confers altruistic or selfish resistance, respectively.

**FIG 2:**
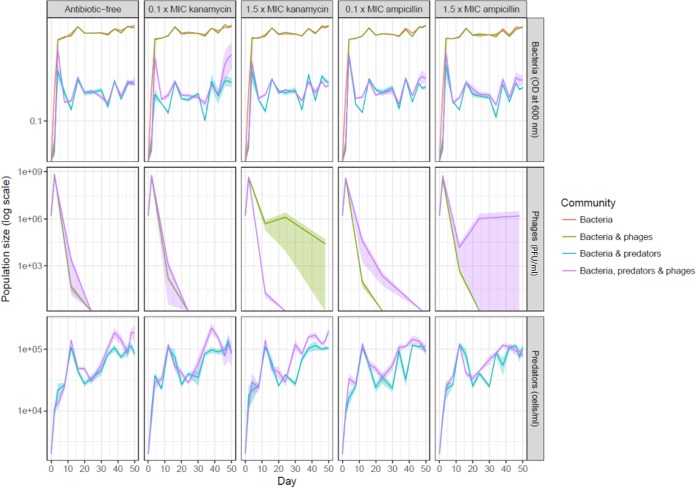
Bacterial, predator, and phage population sizes over time in each treatment in a 50-day microcosm experiment (data represent means ± standard errors [SE]). All treatments were replicated four times.

We used an optical density (OD)-based method, light microscopy, and a plaque assay to track bacterial, protozoan, and phage population sizes, respectively, over time. Bacterial population size was not affected by antibiotic type or concentration but was affected by community composition (linear mixed models [LMM] antibiotics, *χ^2^* = 3.32, df = 2, *P = *0.19; kanamycin concentration, *χ^2^* = 0.44, df = 2, *P = *0.80; ampicillin concentration, *χ^2^* = 3.29, df = 2, *P = *0.19; community composition, *χ^2^* = 4,091.5, df = 42, *P < *0.001) (Fig. 2). Protozoan predation decreased the bacterial population size, regardless of the presence or absence of phage (general linear hypothesis test [glht], predator treatments versus predator-free treatments, *P < *0.001 for all), although the population size was elevated slightly (1.08-fold on average, discounting the transient phase on days 2 to 4) in the simultaneous presence of phage (glht, predator alone versus predator with phage, *P = *0.003).

Similarly to the results seen with bacteria, only community composition affected the protozoan population size, such that ciliate density was increased in the simultaneous presence of phages (LMM antibiotic, χ2 = 2.81, df = 2, *P = *0.25; kanamycin concentration, *χ^2^* = 2.19, df = 2, *P = *0.33; ampicillin concentration, *χ^2^* = 1.77, df = 2, *P = *0.41; community composition, *χ^2^* = 92.14, df = 14, *P < *0.001) (Fig. 2). The phage population size peaked early in the experiment and subsequently decreased to low levels (Fig. 2). Overall population sizes did not differ between the two antibiotic treatments (LMM, *χ^2^* = 3.72, df = 2, *P = *0.16), but the treatments affected the population sizes differently. With kanamycin, concentration and community composition interactively affected phage population size (LMM concentration, *χ^2^* = 64.13, df = 16, *P < *0.001; community composition, *χ^2^* = 63.05, df = 12, *P < *0.00; concentration × community composition, *χ^2^* = 64.13, df = 16, *P < *0.001), such that in the presence of a lethal kanamycin concentration without predation, extinction of the phage population was delayed compared with the results seen with other environments (glht, 1.5× MIC versus 0/0.1× MIC [both], *P < *0.01; 0 versus 0.1× MIC, *P = *0.99). In contrast, with ampicillin, the concentration did not significantly affect the phage population size (LMM, *χ^2^* = 5.96, df = 2, *P = *0.05), and community composition had the opposite effect (LMM, *χ^2^* = 4.32, df = 1, *P = *0.038), such that the phage population size was increased or extinction of phage populations was delayed in the presence of protozoa.

### Leakiness of resistance and ecological interactions determine plasmid persistence more than lethal antibiotic concentrations.

While the altruistic/leaky nature of beta-lactam resistance (in this study, ampicillin resistance) is well studied (27), as is also the case for the strains utilized in this study (28), we first confirmed the selfish/nonleaky nature of aminoglycoside resistance (in this study, kanamycin resistance) provided by plasmid RP4. Therefore, the ability of a conjugation-defective HMS174(RP4) strain to support a susceptible strain in the presence of kanamycin (25 µg ml^−1^ and 50 µg ml^−1^) was measured. No surviving cheaters (i.e. bacteria that did not encode resistance themselves) were observed (*n* = 5).

**FIG 3:**
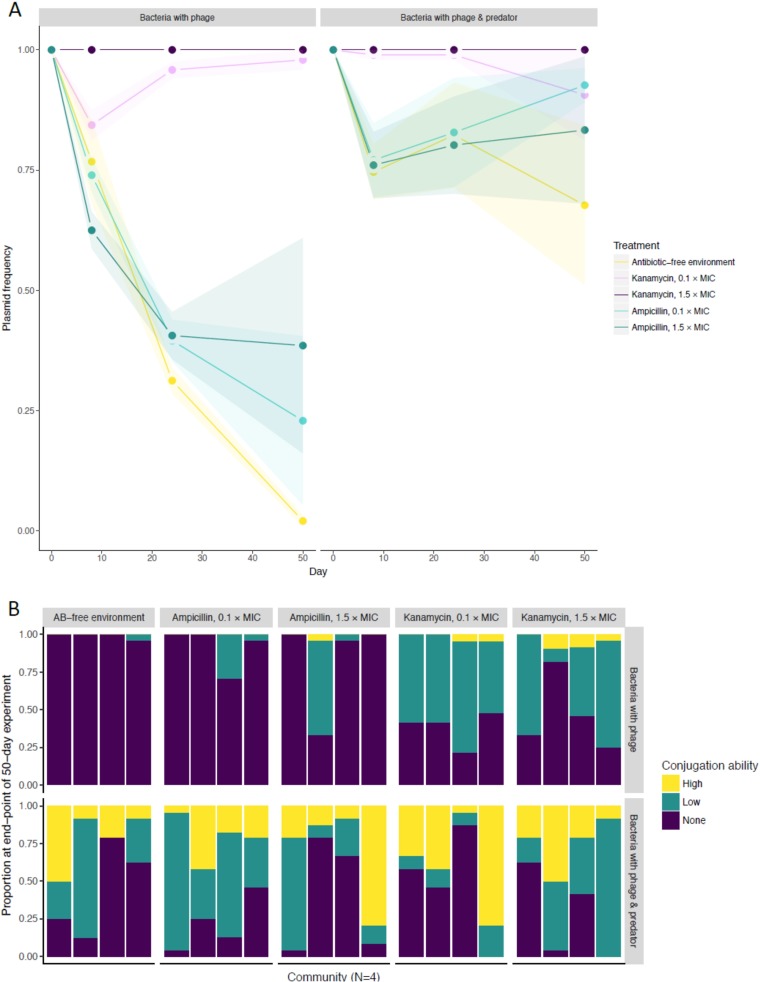
(A) Frequency of plasmid harboring bacteria over the course of a 5-day community experiment in different environments (data represent means ± SE). Plasmid RP4-encoded resistance mechanisms against kanamycin and ampicillin represent selfish and altruistic resistance mechanisms, respectively. Altruism of resistance and protozoan predation, rather than antibiotic concentration, are implicated as major drivers of plasmid persistence in the presence of plasmid-dependent phage. Because plasmid loss was not observed in the absence of phage, only populations from treatments containing phage are shown. Each treatment was replicated four times. (B) Observed conjugation ability for plasmid-harboring clones of bacteria isolated from the end of the community experiment. 24 clones were isolated from all four experiments, and the phenotypes are depicted separately for each replicate. AB, antibiotic.

We determined plasmid prevalence over time by isolating clones from three time points within the 50-day experiment and culturing on agar plates containing high concentrations of selective antibiotics. Plasmid loss was observed only in bacterial populations with plasmid-dependent phages selecting against the plasmid (LMM community composition, *χ^2^* = 306.1, df = 31, *P < *0.001; glht, phage alone versus other community compositions [all comparisons], *P < *0.02) (Fig. 3A). Plasmid loss caused by phages was counteracted by the simultaneous presence of protozoa, such that the plasmid loss results did not significantly differ from those seen with phage-free treatments (glht [all comparisons], *P* = not significant [NS]), despite individual replicate populations exhibiting considerable decreases in plasmid prevalence (Fig. 3A). Similarly, kanamycin―against which the plasmid confers selfish resistance―maintained the plasmid at high prevalence in populations in the presence of phages (LMM antibiotic, *χ^2^* = 335.8, df = 30, *P < *0.001; glht, kanamycin versus ampicillin/antibiotic-free environment, *P < *0.001). In contrast, the plasmid reached a low frequency with ampicillin―against which the plasmid confers leaky (altruistic) resistance. The plasmid loss seen with ampicillin did not differ significantly from that seen with the antibiotic-free environment (glht, ampicillin versus antibiotic-free environment, *P = *0.33). This result potentially is the consequence of high variability between replicate communities, as the mean prevalence reached was 0%, 20%, or 40% under conditions of no, sublethal, or lethal kanamycin selection, respectively, which is consistent with the Black Queen hypothesis of stable coexistence between helpers and beneficiaries. Increasing the antibiotic level from a sublethal to a lethal concentration did not have a significant effect on plasmid persistence with either antibiotic (LMM ampicillin concentration, *χ^2^* = 1.91, df = 2, *P = *0.39; LMM kanamycin concentration, *χ^2^* = 308.4, df = 30, *P < *0.001; glht, kanamycin at 0.1/1.5× MIC versus antibiotic-free environment, *P < *0.001 [but for 0.1 versus 1.5× MIC, *P = *0.92]).

### Plasmid-dependent bacteriophages select for defective conjugation counteracted by conjugation-selecting protozoa.

Phage PRD1 selects for various types of plasmid mutants whose conjugation ability is either reduced or lost (see Fig. S1 in the supplemental material). To measure the loss of wild-type conjugation ability during the 50-day microcosm experiment, we cocultured clones isolated from the end of the experiment with recipient strain E. coli K-12 JM109(pSU19). This was followed by culturing in agar plates containing antibiotics that allow only those recipient bacteria to grow that have acquired the conjugative plasmid from the clone and by rating cultures from 1 to 4, where 1 represents no growth and 4 represents normal growth (i.e. wild-type conjugation ability; see Fig. S2). The persistence of the wild-type plasmid conjugation ability at the end of the evolution experiment was not affected by antibiotic or concentration (beta regression antibiotic, *χ^2^* = 1.00, df = 2, *P = *0.61; kanamycin concentration, *χ^2^* = 1.03, df = 2, *P = *0.60; ampicillin concentration, *χ^2^* = 2.16, df = 2, *P = *0.34) (Fig. 3B). With both kanamycin and ampicillin (beta regression community composition, *χ^2^* = 130.1, df = 3, *P < *0.001), conjugation ability was almost completely lost with phages alone (glht, phage alone versus all other community compositions, *P < *0.001), was decreased but significantly retained under conditions of phage selection by the simultaneous presence of protozoa (glht, phage with protozoa versus bacteria alone or with protozoa, *P < *0.001), and was completely retained with predation alone (glht, bacteria alone versus bacteria with protozoa, *P = *0.85).

10.1128/mSystems.00104-18.2 FIG S1 Plasmid-dependent bacteriophage selects for mutant plasmids that have reduced or abolished conjugation ability as summarized from the work by Ojala et al. (26). The observed mutations had different effects on conjugation ability and responded differently to subsequent selection favoring the reversal of the conjugation ability. Insertions of adjacent sequences (dynamic mutation) within the *trbI* gene reduced the conjugation ability of the plasmid, but the level of conjugation ability was simultaneously readily reversible to the wild-type status under conditions of positive selection for conjugation. Transposon insertions between *trbJ* and *trbL* reduced the conjugation ability, and the ability was not observed to revert to the wild-type status. Deletion in *trbJ* resulted in loss of conjugation, which was not observed to reverse. Download FIG S1, TIF file, 1.0 MB. Copyright © 2018 Cairns et al. 2018 Cairns et al. This content is distributed under the terms of the Creative Commons Attribution 4.0 International license.

10.1128/mSystems.00104-18.3 FIG S2 Experimental setup for studying the conjugation ability of individual clones from community experiments. Isolated clones were cultivated on a 96-well plate and transferred into a plate containing suitable recipient bacteria. Samples of the cocultures were plated on antibiotic plates where only bacteria that had received the plasmid from clones could survive. The “A” in the shield in the cartoon represents the possibility of altruistic rescue of the plasmid-free bacteria. Original images of plasmid conjugation experiments are available in the Dryad depository (doi no. 10.5061/dryad.10gk660). Download FIG S2, PDF file, 1.2 MB. Copyright © 2018 Cairns et al. 2018 Cairns et al. This content is distributed under the terms of the Creative Commons Attribution 4.0 International license.

### Bacterial metabolic activity is elevated under conditions of protozoan predation, potentially promoting conjugation activity and mediating plasmid persistence in bacterial communities.

**FIG 4:**
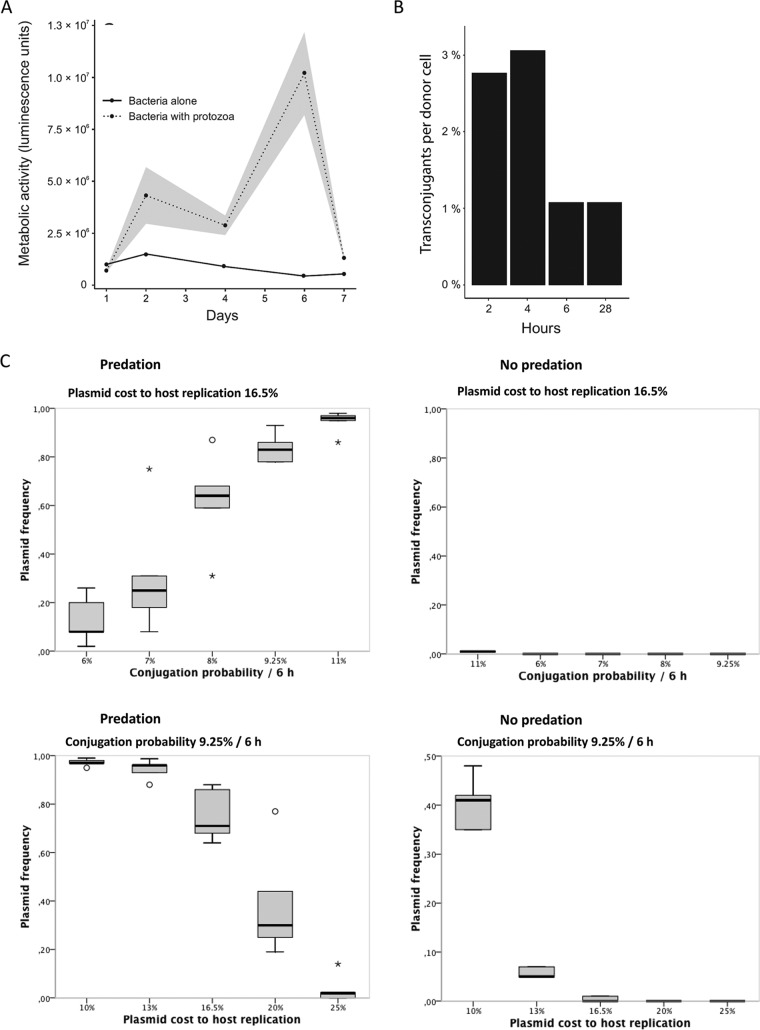
Evidence regarding potential mechanisms by which protozoa maintain plasmid conjugation in bacterial populations. (A) Per-cell metabolic activity in bacterial populations in the presence (dashed line) or absence (solid line) of predation by the protozoan T. thermophila (data represent means ± SE). Both treatments were replicated four times. (B) Bacterial conjugation rates in different growth phases. The early to mid-logarithmic-growth phase is represented by the 2-h and 4-h time points, the late logarithmic-growth phase by the 6-h time point, and the stationary-growth phase by the 28-h time point. The bar height represents the mean of results from five technical replicates. (C) Effects of plasmid cost and conjugation probability on plasmid maintenance in simulated communities with and without predation. Plasmid cost indicates the relative decrease in replication frequency due to plasmid carriage. Bacterial population density was set to modify the conjugation probability. Since predation lowered the effective population density of the community and thus increased the conjugation rate, the prevalence of plasmid increased in the presence of protozoa after disruption of the conjugation network (i.e. the emergence of plasmid-free individuals) (*n* = 5).

We hypothesized that selection for plasmid conjugation in bacterial populations under conditions of protozoan predation might be caused indirectly by lower cell densities under conditions of predation, since low cell densities may maintain higher metabolic activity and, thereby, higher conjugation activity than high cell densities where cells assume the stationary phase. To test this, we conducted a separate 8-day microcosm experiment using a luminescence-based method to measure differences between the relative levels of ATP production by bacterial cells with or without predation. Bacterial metabolic activity per cell was higher in the presence of protozoan predation than in the absence of protozoa (LMM, *χ^2^* = 100.4, df = 5) (*P < *0.001) (Fig. 4A).

In addition, to test whether more-active cells are more likely to conjugate, we determined the conjugation rate of plasmid RP4 in different growth phases of the host bacterium HMS174 by culturing for 2 h, 4 h, 6 h, or 28 h, mixing with the recipient E. coli JM109(pSU19) strain, allowing bacteria to conjugate for 2 h, and plating on medium selective for transconjugated JM109(pSU19)(RP4) cells. The conjugation rates of the wild-type HMS174(RP4) plasmid differed depending on the growth phase (Fig. 4B). Each cell in a bacterial culture in five independent experiments that had been growing for 2, 4, 6, and 28 h conjugated (on average) with a probability of 2.8%, 3%, 1.1%, and 1.1%, respectively.

To further evaluate the influence of predation on plasmid persistence, an individual-based model was constructed (Fig. S3). In this model, populations containing both plasmid-harboring and plasmid-free bacteria were exposed to differing environmental conditions. Conjugation probability was adjusted based on the population density in the system such that the probability decreased from the wild-type probability level to one-third that level in relation to population density. The generation time and conjugation probability were adjusted based on the microcosm experiment, where around eight bacterial generations corresponded to a 48-h culture refreshment cycle (see Table S1 in the supplemental material). The fitness cost determined for plasmid carriage was based on the replication rates measured in the absence of antibiotics (Table S2). Similarly to *in vitro* experiments, simulated predation significantly improved plasmid survival over 175 generations (iterations of the model) by modulating the bacterial population size (Fig. 4C). While plasmid frequencies in simulations align with the observed frequencies in serial culture experiments when experimentally determined parameter values are used, the plasmid nevertheless disappears from the system when a much greater number of generations (>10^4^) is simulated. This suggests that plasmid-host coadaptation and selective sweeps (such as the transient presence of antibiotics) may be necessary for ensuring the long-term survival of the plasmid in real-life settings even in the presence of predators. To investigate this, we explored how different factors influence plasmid persistence. Indeed, when the plasmid-associated fitness cost was decreased from the observed 16.7% to just 15%, the plasmid occasionally (3 of 5 individual simulations) survived for over 10^4^ generations (example simulations shown in Fig. S4). Also, exposure to 0.1× MIC antibiotics (against which the plasmid encodes nonleaky resistance) for 10 simulation cycles in every 100 cycles significantly improved plasmid survival (5 of 5 simulations) (Fig. S4C).

10.1128/mSystems.00104-18.4 FIG S3 Depiction of the actors in the individual-based model and the factors that influence their frequency in the community. B_WT_, bacterium with wild-type conjugation ability; B_RED_, bacterium with reduced conjugation ability; B_DEF_, bacterium with defective conjugation ability; B_F_, plasmid-free bacterium; Phage, plasmid-dependent bacteriophage. Download FIG S3, TIF file, 0.05 MB. Copyright © 2018 Cairns et al. 2018 Cairns et al. This content is distributed under the terms of the Creative Commons Attribution 4.0 International license.

10.1128/mSystems.00104-18.5 FIG S4 Examples of results of plasmid survival over 10,000 simulation cycles (∼generations, *x* axis). Panels A and B differ only by the associated plasmid cost. In the experiment represented in panel C, a simulated bacterial community was exposed to 0.1× MIC antibiotic selection for 10 cycles in every 100 no-selection cycles. Download FIG S4, TIF file, 0.2 MB. Copyright © 2018 Cairns et al. 2018 Cairns et al. This content is distributed under the terms of the Creative Commons Attribution 4.0 International license.

10.1128/mSystems.00104-18.6 TABLE S1 Model parameters, their effects, and values used. Download Table S1, DOCX file, 0.01 MB. Copyright © 2018 Cairns et al. 2018 Cairns et al. This content is distributed under the terms of the Creative Commons Attribution 4.0 International license.

10.1128/mSystems.00104-18.7 TABLE S2 Growth parameters for E. coli K-12 (HMS174) with and without conjugative plasmid RP4 in 0.1× MIC antibiotics (Km, kanamycin; Ap, ampicillin) and without antibiotics. Carrying capacity is measured as optical density (OD) at 600 nm. Growth rate and generation time are expressed in per-hour values. Download Table S2, DOCX file, 0.01 MB. Copyright © 2018 Cairns et al. 2018 Cairns et al. This content is distributed under the terms of the Creative Commons Attribution 4.0 International license.

## DISCUSSION

In this study, we investigated the maintenance of a conjugative plasmid providing leaky (altruistic) and nonleaky (selfish) resistance against beta-lactams and aminoglycosides, respectively, in a multitrophic system consisting of bacterial prey, plasmid-dependent bacteriophage, and protozoan predator. The plasmid-dependent bacteriophage PRD1 was used to disrupt the conjugation network, and, indeed, its presence alone caused E. coli populations to lose their plasmids, thus rendering them susceptible to antibiotics. The altruistic nature of beta-lactam resistance was clearly seen when the phage-bacterium combination was cultivated in ampicillin as only a subpopulation of bacteria retained the plasmid. In terms of Black Queen evolution, this is a clear example of a race to the bottom, as the benefits of plasmid carriage were distributed among the community members but the cost was borne by individuals, thus favoring beneficiaries over helpers. In contrast to the ampicillin results, the presence of kanamycin caused the entire population to maintain the plasmid over the 50-day period, likely owing to the nonleaky nature of the resistance mechanism. Surprisingly, there were only minor differences between the results seen with lethal (1.5× MIC) and sublethal (0.1× MIC) antibiotic levels, suggesting that leakiness of resistance rather than antibiotic concentration is the defining factor in plasmid maintenance. Bottery and colleagues have observed similar outcomes with selfish tetracycline resistance (29).

While antibiotics forced plasmid maintenance, bacteriophage selection still caused the vast majority of the plasmids to become conjugation defective. This phenotype was retained over the course of the experiment, disregarding the fact that phages rapidly disappeared from most of the communities. Notably, however, selection for selfish resistance hindered the complete loss of plasmid-dependent phage from the community in comparison to selection for altruistic resistance and to the absence of antibiotics, as the phage were present also at the end of the experiment. The addition of the protozoan predator T. thermophila to the system had a major impact on the frequency of plasmids maintaining a conjugative phenotype in both the absence and presence of antibiotics. This is in line with previous experiments denoting the importance of predation for conjugative plasmid persistence (24). Since a bacterial population reaches lower density under conditions of predation (Fig. 2), it is possible that the individual cells remain in a more active state throughout each step of the serial culture experiment, which, in turn, increases the dissemination of conjugative plasmids between individual bacteria. We investigated this further by showing that the bacteria conjugated approximately three times more frequently in the early logarithmic-growth phase than in the late logarithmic-growth and stationary-growth phases. Also, the metabolic activity of bacteria was shown to remain significantly higher in the presence of T. thermophila, with all of the results suggesting that predation has an indirect influence on the conjugation rate.

Lopatkin and colleagues demonstrated that the absence of antibiotic selection alone is not enough to cause bacterial communities to become sensitive to antibiotics (1). Yet they also showed that plasmid-mediated resistance is more likely to be lost if the conjugation network is disrupted by chemical agents. We observed here that a similar outcome can be achieved with biological agents, namely, plasmid-dependent phages. However, even subinhibitory concentrations of antibiotics can have a notable impact on plasmid prevalence, depending on the leakiness of the plasmid-encoded resistance mechanism. Simulations also suggested that the long-term survival of plasmids may depend on a minor decrease in the plasmid-associated fitness cost and/or periodical selection. Hence, bacterium-plasmid coadaptation and fluctuations in environmental conditions are factors that might nullify the outcome of resensitization applications which target the conjugation network. Even more intriguing is the notion that predation can effectively counter the loss of resistance plasmids after the disruption of the conjugation network. Since predation selects for conjugative plasmids over nonconjugative ones (24), and given that conjugation may be lost by several types of mutations, of which only some are readily reversible (see Fig. S1 in the supplemental material), under oscillating selection pressures (for and against conjugation), mutations in these reversible sites could be considered contingency loci improving the survival of resistance plasmids. Therefore, taking trophic interactions into account in real-life systems may play a defining role in whether or not resistance is actually lost from the community.

Nevertheless, predation may not be of particular relevance in human carriage of resistance plasmid-harboring bacteria, at least not in Western societies where intestinal protozoa are rare among individuals (23). On the other hand, in many developing countries in sub-Saharan Africa and Southeast Asia, the majority of children (especially in slums) are infected with protozoa such as Entamoeba histolytica by the age of 2 years (30). These countries also experience a severe burden of antibiotic-resistant bacteria and resistance-associated effects on health care (31). It is possible that the trophic interplay between eukaryotic and prokaryotic microbes might be also furthering the overall persistence of resistant bacteria in these areas. However, other environments with low concentrations of antibiotics, such as farms and wastewater treatment plants, may be habitats where the role of predation in resistance maintenance is likely to be more prominent.

## MATERIALS AND METHODS

### Strains and culture conditions.

We used E. coli K-12 HMS174 as the bacterial host species (32). MICs of antibiotics were determined with the plasmid-free ancestral strain, and for the community experiment, the plasmid RP4 was transconjugated to HMS174 from E. coli K-12 JE2571 (33). Broad-host-range conjugative plasmid RP4 (incompatibility group P) has multiple genes for addicting the host and encodes resistance to the antibiotics kanamycin, ampicillin, and tetracycline (34). We used virulent double-stranded DNA (dsDNA) plasmid-dependent bacteriophage PRD1 (family *Tectiviridae*) (35) as the viral parasite and the ciliate Tetrahymena thermophila CCAP 1630/1U (axenic stock obtained from Culture Collection for Algae and Protozoa, United Kingdom) as the protozoan predator.

We followed previously established protocols for microcosm experiments with bacterium-phage and bacterium-ciliate systems (36–42). The culture medium for bacteria contained M9 salts and King’s B (KB) nutrients at a 5% concentration compared to full-strength medium (concentrations used, 1 g peptone number 3 and 0.5 ml of 85% glycerol in 1 liter of dH_2_O). All media and microcosm vials were sterilized by autoclaving prior to use and kept at 28°C (±0.1°C) during the experiments, with constant rotation at 50 rpm. In the conjugation ability and rate experiments, we used lysogeny broth (LB) medium (43) and E. coli K-12 JM109 harboring chloramphenicol resistance-encoding plasmid pSU19 (44) as the recipient strain.

### Community experiment.

In order to determine antibiotic MIC values for the community experiment, the ancestral HMS174 strain was cultured under experimental conditions in a concentration gradient of 0 to 1.9 µg ml^−1^ ampicillin or 0 to 4.5 µg ml^−1^ kanamycin (19 different concentrations with both antibiotics). Bacterial growth was measured as optical density (Bioscreen C spectrophotometer; Oy Growth Curves Ab Ltd.) using a 420-to-580-nm wideband filter, and the MIC was determined as the lowest concentration with no detectable bacterial growth after 96 h. The MICs were 1.1 µg ml^−1^ and 2.5 µg ml^−1^ for ampicillin and kanamycin, respectively.

To test for the interactive and relative contributions of trophic interactions, altruism of resistance, and antibiotic concentration to plasmid persistence in HMS174(RP4) with different concentrations and types of antibiotic, we performed a 50-day microcosm experiment. We used a community treatment consisting of the presence or absence of phage or protozoan and an antibiotic treatment consisting of no antibiotic, 0.1× MIC of ampicillin (0.11 µg ml^−1^) or kanamycin (0.25 µg ml^−1^), or 1.5× MIC of ampicillin (1.65 µg ml^−1^) or kanamycin (3.75 µg ml^−1^). All treatments were started from a clonal culture of HMS174(RP4) cultured overnight in KB. The initial bacterial density was approximately 5.4 × 10^6^ CFU ml^−1^ and the initial phage density approximately 1.6 × 10^6^ PFU ml^−1^, constituting a multiplicity of infection (MOI) value of 0.3. The initial protozoan density was 2 × 10^3^ cells ml^−1^. All treatment combinations were replicated four times in 25-ml glass vials containing 6 ml KB. Every 48 h, 1% (60 µl) of each culture was transferred to a new vial containing fresh KB. Every 96 h (or every 48 h for the first three transfers), bacterial density was estimated as optical density (OD) at 600 nm (UV-1800 spectrophotometer; Shimadzu, Japan), and T. thermophila density was enumerated directly from live samples using a compound microscope (Zeiss Axioskop 2 plus; Oberkochen, Germany), as described previously (36). A 1.0-ml subsample was frozen with 0.5 ml of 85% glycerol or without glycerol (for phage analyses) and kept at –80°C for later analysis. Phage abundances were estimated for days 2, 12, 24, and 48 from freeze-stored samples using plaque assay (44).

### Plasmid persistence.

To detect the loss of RP4 plasmid during the community experiment, we isolated 24 bacterial clones per population from freeze-stored samples from day 8, the middle (day 24), and the endpoint (day 50) of the experiment. Clones were inoculated in 200 µl of LB medium in a 96-well plate, cultured overnight, and frozen with 50 µl of 87% glycerol at –80°C for later analysis. To test for the presence of plasmid, a 10-µl subsample was cryo-replicated (45) on a large (140-mm-diameter) petri dish containing LB agar with high concentrations of all antibiotics to which the RP4 encodes resistance as follows: 150 µg ml^−1^ ampicillin, 25 µg ml^−1^ kanamycin, and 20 µg ml^−1^ tetracycline.

### Bacterial cheaters in the presence of aminoglycoside kanamycin.

We investigated the capacity of JM109(pSU19) to survive at lethal concentrations of ampicillin (150 µg ml^−1^ and 300 µg ml^−1^) or kanamycin (25 µg ml^−1^ and 50 µg ml^−1^) in the presence of a conjugation-deficient HMS174(RP4) mutant. The conjugation-deficient mutant was created as described previously (26). Briefly, HMS174(RP4) was cultured overnight (37°C, 220 rpm) in the presence of kanamycin and plasmid-dependent phage PRD1 and plated on LB agar. Several clones were picked, and their ability to conjugate with JM109(pSU19) was investigated. A mutant producing no transconjugants was selected for the cheating experiment. Subsequently, 5-µl volumes of overnight cultures of JM109(pSU19) and HMS174(RP4) mutants were cultured together in 5 ml of LB medium with antibiotics at different concentrations for 21 h (37°C, 220 rpm). These cultures were plated on chloramphenicol (25 µg ml^−1^) to select for JM109(pSU19) cells that had survived in the presence of ampicillin or kanamycin.

### Conjugation ability.

In order to measure the conjugation ability of bacteria at the endpoint of the evolution experiment (day 50), clones were isolated and transferred to 200 µl of KB medium in 96-well plates. Another 96-well plate was prepared with recipient strain E. coli K-12 JM109(pSU19). Both plates were cultured overnight at 37°C. A third 96-well plate was prepared with 200 µl of LB medium in each well. A plate replicator was used to transfer the clones from evolution experiments with the recipient bacterium to the third plate. This conjugation plate was cultured overnight at 37°C. A plate replicator was utilized to transfer samples from conjugation experiments to large petri dishes containing antibiotics that allowed only those recipient bacteria to grow that had acquired the conjugative plasmid from the clone. Petri dishes were transferred to 37°C conditions, and conjugation ability was inferred based on overnight growth in sampled wells. Each spot was assigned to one of the following three categories: (i) no growth, (ii) some growth, and (iii) normal growth (where “no growth” indicates complete loss of conjugation ability and “normal growth” indicates wild-type conjugation ability) (see Fig. S2 in the supplemental material).

### Bacterial metabolic activity.

To test for the effect of protozoan predation on bacterial metabolic activity, we conducted an 8-day experiment using a luminescence-based method to measure differences between the relative levels of production of ATP by bacterial cells with or without predation. The test was started from an overnight clonal culture of HMS174(RP4). The same initial bacterial and predator densities as those described for the community experiment were inoculated into 50 ml of experimental medium (5% KB) in a 250-ml screw-cap polyethylene terephthalate (PET) storage bottle (Corning, New York, USA), with two treatments: bacteria with live predator and bacteria with heat-killed predator (to eliminate any effect of predator cells or carryover medium). Both treatments were replicated four times. Culturing was performed for 8 days without transfers or rotation.

On days 1, 2, 3, 4, 6, and 8, aliquots of 2 ml were filtered through 5-µm-pore-size filters to remove ciliates. The filtrates were used to measure bacterial density (36) and metabolic activity (ATP production). Metabolic activity was measured with a well plate reader (Victor3 1420 Multilabel Counter; PerkinElmer, MA, USA) using the BacTiter-Glo microbial cell viability assay (Promega, Madison, WI, USA) according to manufacturer’s instructions, except that the culture and BacTiter-Glo reagent were mixed at a 2:1 ratio instead of a 1:1 ratio (comparable results were observed). Bacterial metabolic activity was estimated as per-cell ATP production. Results were corrected for nonlinearity (a slight increase in per-cell ATP signal with decreasing cell density). The luminescence signals of sterile medium and filtered ciliate stock did not differ, demonstrating that the filtration had removed any effect of the ciliates on the results.

### Conjugation rate in different growth phases.

We determined the conjugation rate of plasmid RP4 at different growth phases of the host bacterium HMS174. HMS174(RP4) and recipient JM109(pSU19) were cultured overnight (37°C, 200 rpm). Subsequently, 5 µl of HMS174(RP4) was transferred to 5 ml of fresh LB medium and cultured at 37°C with constant rotation at 200 rpm. A 5-µl subsample was taken from this culture after 2 h, 4 h, 6 h, and 28 h and combined with the recipient bacterium at a 1:20 ratio along with the addition of 100 µl of LB. The bacteria were allowed to conjugate for 2 h, after which they were plated on LB agar containing 150 µg ml^−1^ ampicillin, 25 µg ml^−1^ kanamycin, and 25 µg ml^−1^ chloramphenicol to select for transconjugated JM109(pSU19)(RP4) cells. Bacterial density was measured for bacteria before the conjugation experiment to determine the ratio of transconjugants per donor bacterium.

### Brief model description.

An individual-based model was constructed in an attempt to investigate whether the effect of predation on plasmid persistence can be replicated *in silico* and to what extent different variables influence the dynamics within the system. The source code for the model is freely available on Dryad, and a more detailed description is presented in Text S1 in the supplemental material. The model consists of the following interacting “biological” entities: bacteria, plasmids, plasmid-dependent bacteriophages, and protozoa (model entities and factors that influence their abundance are depicted in Fig. S4). Bacteria replicate in the system for as long as the environment maintains the carrying capacity. Each bacterium has an individual probability of being replicated during a single iteration of the model. The standard probability of 1.0 is lowered with plasmid carriage (owing to plasmid-associated fitness costs). The conjugation rate is adjusted based on bacterial population density such that as the population approaches the carrying capacity of the system, the conjugation rate decreases to one-third of the maximum rate. A protozoan requires a preset number of bacteria to be consumed before it can replicate, and a protozoan consumes a fixed number of bacteria during each iteration of the simulation. Aminoglycoside-like antibiotics can be introduced into the simulated community (using either constant or periodic exposure) (e.g., 0.1× MIC antibiotic kills the bacterium with a 10% probability). All parameters can be adjusted by the user. The values used in this study are listed in Table S1 in the supplemental material.

10.1128/mSystems.00104-18.1 TEXT S1 Detailed description of the individual-based model. Download Text S1, DOCX file, 0.01 MB. Copyright © 2018 Cairns et al. 2018 Cairns et al. This content is distributed under the terms of the Creative Commons Attribution 4.0 International license.

### Statistical analyses.

For the 50-day microcosm experiment, we conducted three analyses each for plasmid prevalence over time, population sizes over time, and proportion of wild-type conjugating plasmids at the end of the experiment. These consisted of separate analyses for kanamycin and ampicillin as well as a combined analysis, disregarding concentration, to compare antibiotics results. All statistical analyses were performed in R v. 3.2.2. For correlations between plasmid prevalence or population size over time and experimental treatments (antibiotic/concentration, community composition, and time) in the 50-day experiment and between ATP production and the predation (presence/absence) treatment in the 8-day experiment, we used lme4 (46) to generate linear mixed models (LMM), with the treatment as the fixed effect and transfer within replicates as the random effect. Models with and without the fixed effect were compared to determine the significance of the correlations. To compare proportions of wild-type conjugating plasmids between treatments at the end of the experiment (day 50), we performed beta regression with the logit link function, which accommodates continuous proportion data, using betareg (47). Multiple comparisons were performed using the general linear hypothesis test (glht, function) in multcomp (48) with default parameters for each model type (i.e., custom *post hoc* contrasts testing whether pairwise differences differ significantly from 0).

### Data availability.

Data are available on the Dryad depository (https://doi.org/10.5061/dryad.10gk660). Data include the population sizes and plasmid frequencies from the evolution experiment, model files, and images from plasmid conjugation ability experiments.
