# Supplementary figures and images for: Black Queen Evolution and Trophic Interactions Determine Plasmid Survival after the Disruption of the Conjugation Network
Source: mSystems. 2018 Oct 2;3(5):e00104-18. doi: 10.1128/mSystems.00104-18 (PMC6172774; doi:10.1128/mSystems.00104-18)

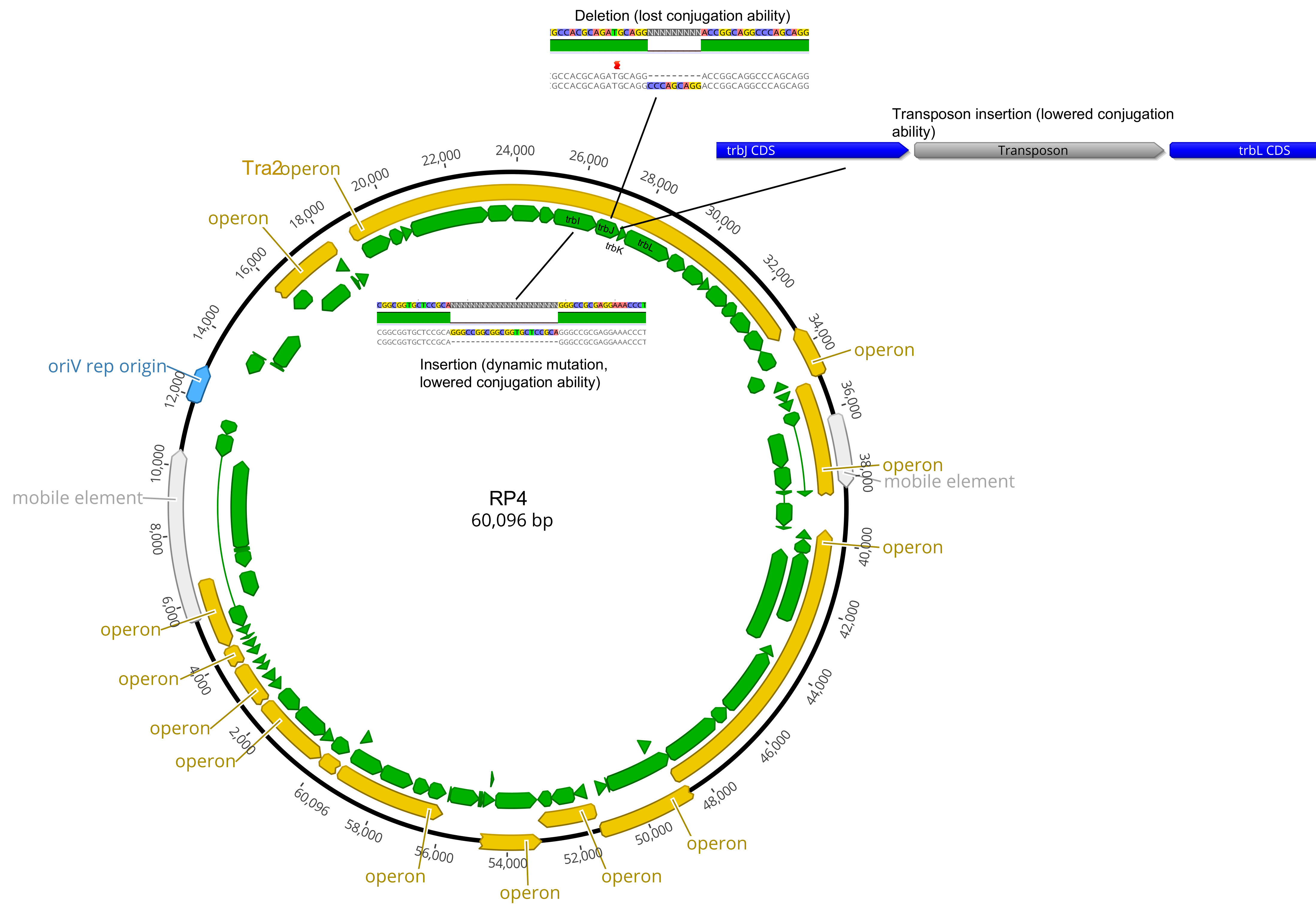

Supplement: FIG S1 [file sys005182268sf1.tif]

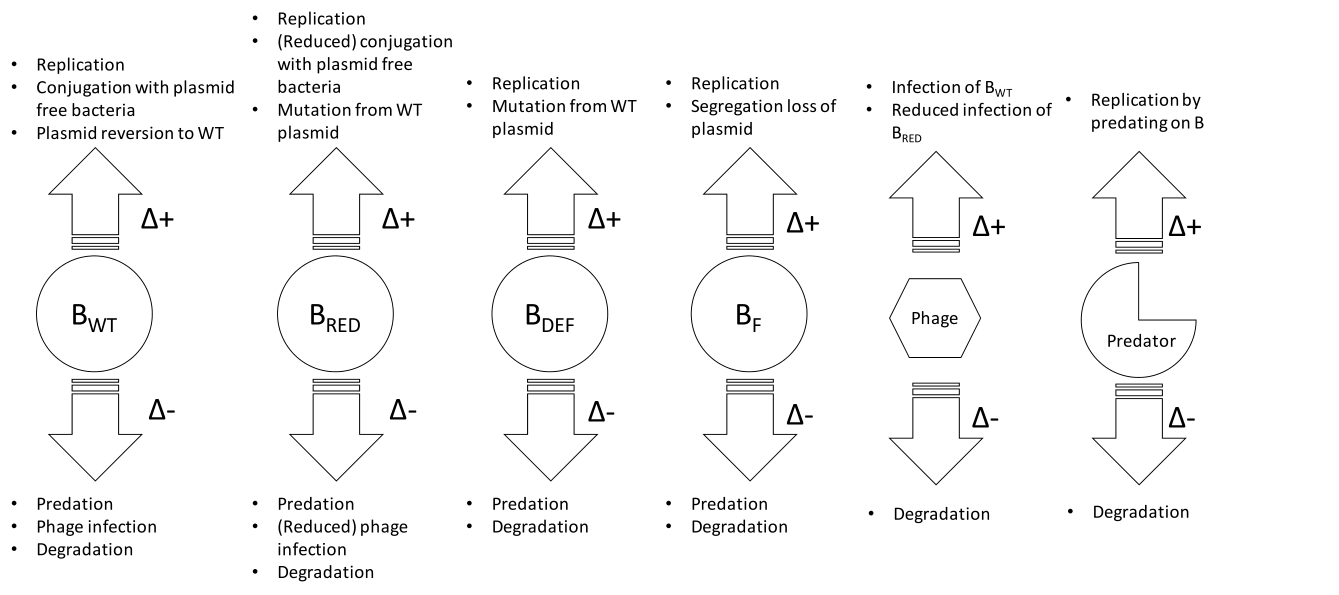

Supplement: FIG S3 [file sys005182268sf3.tif]

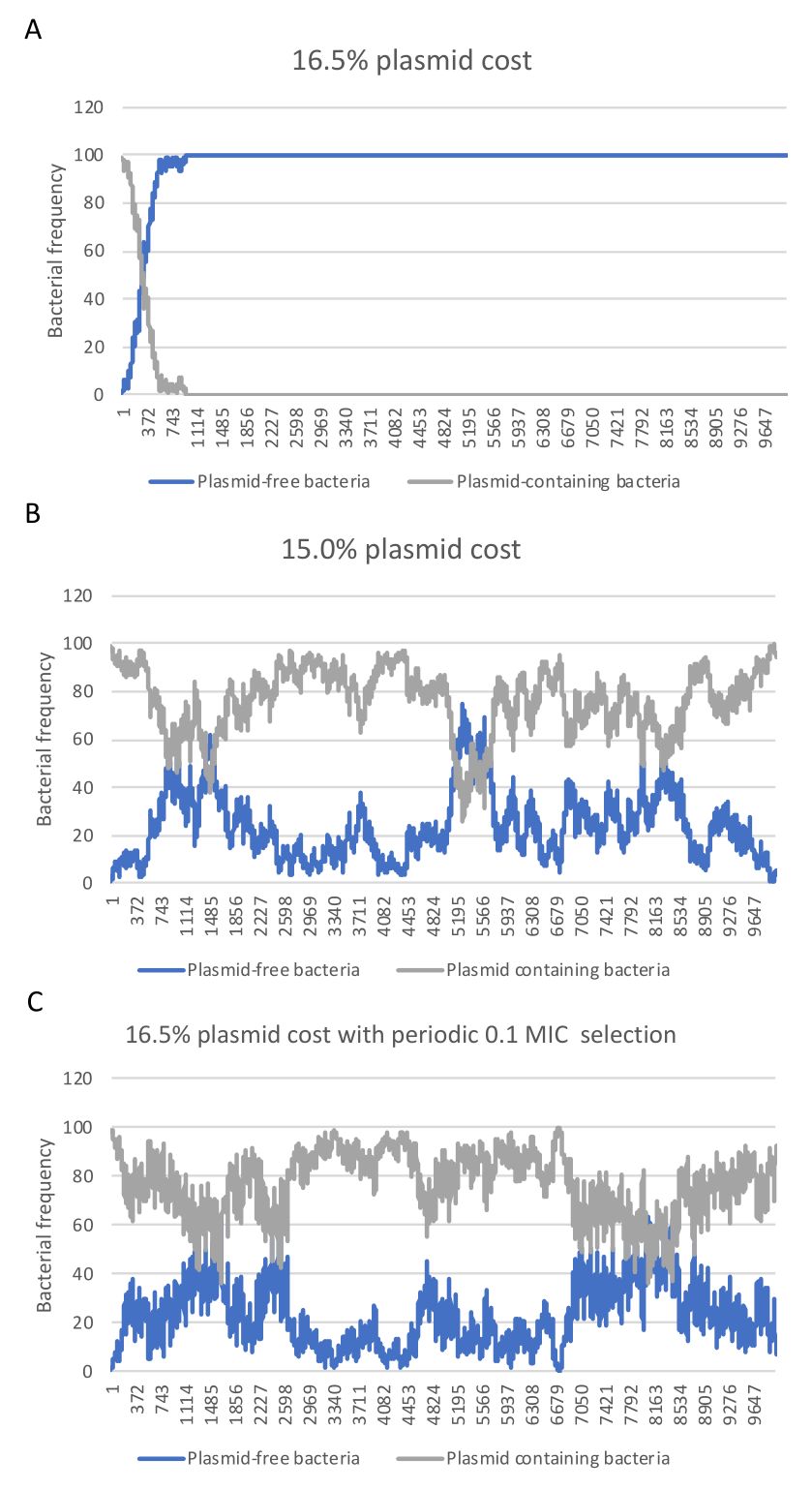

Supplement: FIG S4 [file sys005182268sf4.tif]
