# Supplementary material for: Black Queen Evolution and Trophic Interactions Determine Plasmid Survival after the Disruption of the Conjugation Network
Source: mSystems. 2018 Oct 2;3(5):e00104-18. doi: 10.1128/mSystems.00104-18 (PMC6172774; doi:10.1128/mSystems.00104-18)
Supplement: FIG S2 [file sys005182268sf2.pdf]

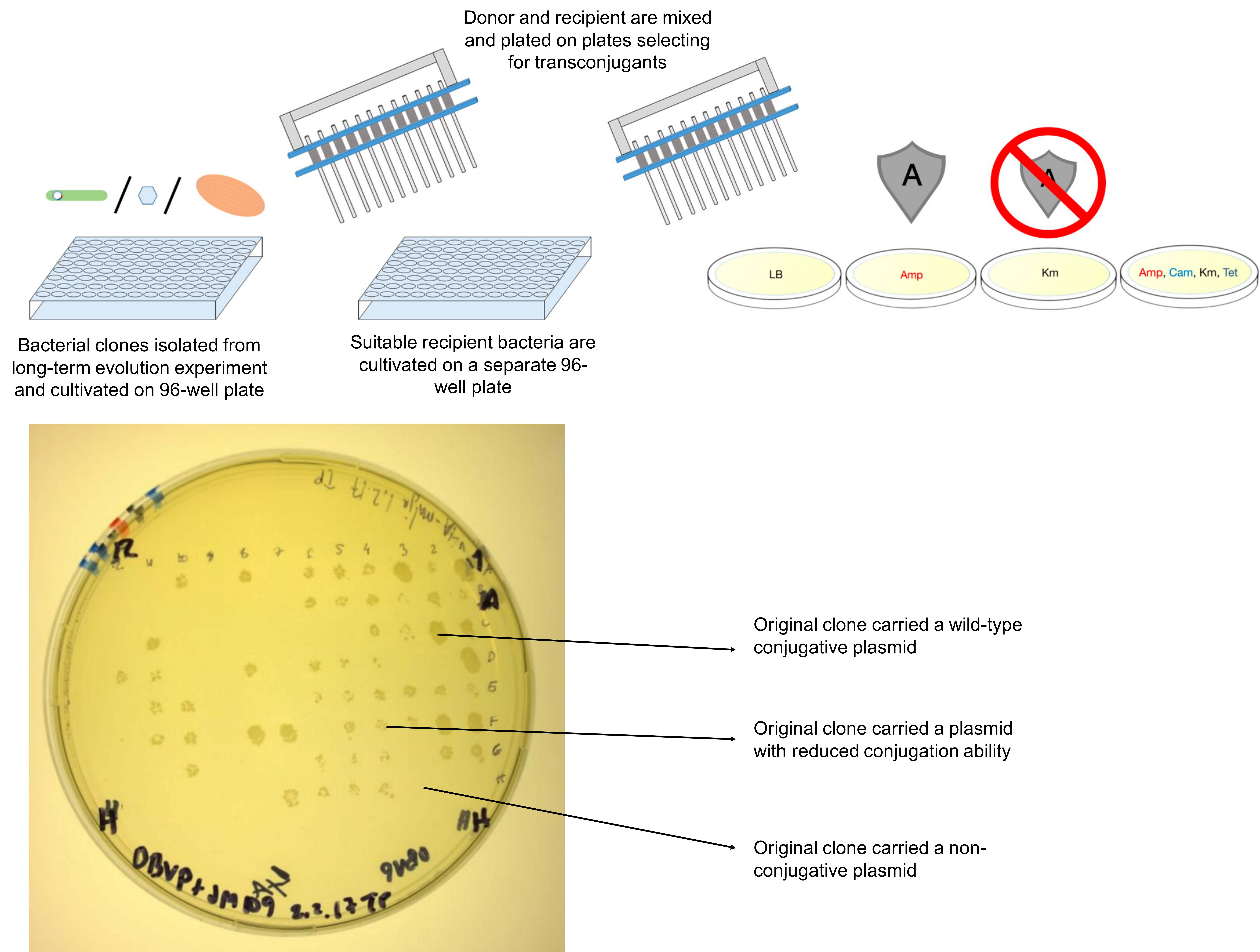
Donor and recipient are mixed and plated on plates selecting for transconjugants
A
LB
Amp
Km
Amp, Cam, Km, Tet
Bacterial clones isolated from long-term evolution experiment and cultivated on 96-well plate
Suitable recipient bacteria are cultivated on a separate 96-well plate
Original clone carried a wild-type conjugative plasmid
Original clone carried a plasmid with reduced conjugation ability
Original clone carried a non-conjugative plasmid
