# Supplementary material for: Black Queen Evolution and Trophic Interactions Determine Plasmid Survival after the Disruption of the Conjugation Network
Source: mSystems. 2018 Oct 2;3(5):e00104-18. doi: 10.1128/mSystems.00104-18 (PMC6172774; doi:10.1128/mSystems.00104-18)
Supplement: TABLE S1 [file sys005182268st1.docx]

**Supplementary Table S1.** Model parameters, their effects and used values.

| Parameter (as in source code) | Effect | Value(s) |
| --- | --- | --- |
| max_population_size | Carrying capacity of the system | 10^5^ |
| protozoa | Amount of protozoa | 1.5 × 10^3^ |
| bacteria | Amount of bacteria | 10^5 |
| wo_plasmid | Percentage of bacteria without a plasmid | 1% |
| w_non_conjugative_plasmid | Percentage of bacteria with conjugation-defective plasmids | 1% |
| w_slowly_conjugating_plasmid | Percentage of bacteria harboring plasmids with reduced conjugation rate | 1% |
| w_wt_plasmid | Percentage of bacteria with wild-type plasmids | 97% |
| plasmid_cost | Reduced replication probability due to plasmid carriage | Varies |
| wt_conjugation | Probability for a plasmid to conjugate (reduced probability if population is approaching carrying capacity of the system) | Varies |
| slow_conjugation | Conjugation frequency of plasmids with reduced conjugation rate | 1/10 of WT rate |
| seg_loss | Probability to lose a plasmid during replication | 0.1% |
| mutate_plasmid | Probability for a plasmid to mutate during replication | 0.1% |
| reversion_wt_plasmid | Reversion to wt plasmid | 0.01% |
| bacteria_degradation | Outflow mortality during each iteration | 5% |
| protozoa_degradation | Outflow mortality during each iteration | 5% |
| predation_per_iteration | Number of bacteria each protozoa consumes during an iteration of the model | 4 |
| feeding_constant | Number of bacteria required to be consumed by protozoa before it replicates | 35 |
