# Supplementary material for: Black Queen Evolution and Trophic Interactions Determine Plasmid Survival after the Disruption of the Conjugation Network
Source: mSystems. 2018 Oct 2;3(5):e00104-18. doi: 10.1128/mSystems.00104-18 (PMC6172774; doi:10.1128/mSystems.00104-18)
Supplement: TABLE S2 [file sys005182268st2.docx]

**Supplementary Table 2.** Growth parameters for E. coli K-12 (HMS174) with and without conjugative plasmid RP4 in 0.1 MIC antibiotics (kanamycin = Km, ampicillin = Ap) and without antibiotics. Carrying capacity is measured as optical density (OD) at 600 nm. Growth rate and generation time are h^-1^.

| Sample | Carrying capacity (*K*) | Growth rate (*r*) | Generation time (hours) |
| --- | --- | --- | --- |
| HMS174 | 0.131 | 0.897 | 0.772 |
| HMS174 (RP4) | 0.132 | 0.747 | 0.928 |
| HMS174 (0.1 × Km) | 0.121 | 0.854 | 0.811 |
| HMS174 (RP4) (0.1 × Km) | 0.120 | 0.662 | 1.0473 |
| HMS174 (0.1 × Ap) | 0.134 | 0.777 | 0.892 |
| HMS174 (RP4) (0.1 × Ap) | 0.124 | 0.732 | 0.947 |
