# Supplementary material for: Black Queen Evolution and Trophic Interactions Determine Plasmid Survival after the Disruption of the Conjugation Network
Source: mSystems. 2018 Oct 2;3(5):e00104-18. doi: 10.1128/mSystems.00104-18 (PMC6172774; doi:10.1128/mSystems.00104-18)
Supplement: TEXT S1 [file sys005182268s1.docx]

**Supplementary material**

**Black Queen evolution and trophic interactions determine plasmid survival after the disruption of conjugation network**

Johannes Cairns^1^, Katariina Koskinen^2^, Reetta Penttinen^2^, Tommi Patinen^2^, Anna Hartikainen^1^, Roosa Jokela^1^, Liisa Ruusulehto^1^, Sirja Viitamäki^1^, Sari Mattila^2^, Teppo Hiltunen^1^ and Matti Jalasvuori^2, 3^

^1^Department of Food and Environmental Sciences, Microbiology and Biotechnology, P.O. Box 65, 00014 University of Helsinki, Finland.

^2^Department of Biological and Environmental Science, Centre of Excellence in Biological Interactions, Nanoscience Center, P.O. Box 35, 40014 University of Jyväskylä, Finland.

^3^Department of Genetics, University of Cambridge, 20 Downing Pl, Cambridge CB2 3DY, UK.

**Supplementary text**

An individual based model was constructed utilizing Java (Sun Microsystems Ltd) programming language. The source code is available via Dryad (doi:10.5061/dryad.10gk660) and it contains several additional features not utilized in this model. The model consists of the following biological entities: bacteria, plasmids, predator (protozoa) and bacteriophages. Bacteria can harbour three different types of plasmids: wild-type conjugative plasmid, slowly conjugating plasmid (reduced conjugation rate and reduced susceptibility to phage infections) and conjugation defective plasmid (no conjugation, resistant to phage infections). All plasmid types can mutate into one of the other types during the replication of bacteria and the probability can be set by the user. Bacteria can also be without a plasmids, and plasmid harbouring bacteria can lose the plasmids due during replication with a user-defined probability. Bacteria harbouring conjugative plasmids has a probability to transfer its plasmid to plasmid-free bacteria during an iteration of the simulation. In this model, a random bacterium is picked from the population and, if it does not harbour a plasmid, then conjugation occurs. If the bacterium already has a plasmid, then nothing happens. Each bacterium can conjugate only once during an iteration. Conjugation rate is modulated by the bacterial density in relative to the carrying capacity of the system. The rate is decreased by arbitrarily selected equation (1) as this equation provides a rapid decline of the rate into 1/3 of the wild type rate when the population density approaches the carrying capacity of the system.

1. ${Conjugation probability=WT_{probability}\cdot(1-\left( \frac{P}{Pmax} \right)}^{10} \cdot\frac{3}{2})$, where *WT_probability_* is the user-defined conjugation probability, *P* is the current population size and *P_max_* is the maximum population size

Bacteria replicate with a user defined probability during each iteration. Resources are not modelled, so replication depends only on the probability. If the system is already in the carrying capacity, then no replication occurs. Bacteria harbouring plasmids have a user-defined decrease in the replication probability (i.e. plasmid associated fitness cost). Resistance can also be set to be costly in itself, indicating that if antibiotics are present, the replication rate is decreased relative to the MIC concentration of the antibiotic.

Bacteria harbouring conjugative plasmids are susceptible to phage infections. There are user defined probability for a phage to recognize a plasmid-harboring bacterium from the community. If infection occurs, then bacteria is removed from the system and a user-defined number of new phages are added.

Predators consume a number of bacteria during each iteration of the model and when enough bacteria has been consumed, a new predator is added to the system. All numbers can be defined by the user. Protozoa do not discriminate between plasmid-harbouring and plasmid-free bacteria. The outflow rates can be set individually for all biological entities. This set percentage is removed from the system by randomly selecting the individuals during each iteration of the model. The system can also be set to be refreshed in user-defined intervals, indicating that only during refreshment cycles the bacteria, predators and phages are removed.

The system can be set to contain antibiotics. The level of antibiotics is set in relative to minimum inhibitory concentration (i.e. 0.1MIC indicates that 10% of bacteria without plasmids are removed from the system due to antibiotic selection during every iteration of the model). The antibiotic can also be set to be present only in user-defined intervals (periodic antibiotic selection). The model can be freely adapted for other modelling purposes by the academic community, if anyone wishes to do so.
